# Minute/instant-MOFs: versatile, high quality, ultrafast, scalable production at room temperature†

**DOI:** 10.1039/c9na00350a

**Published:** 2019-08-02

**Authors:** Vijayan Srinivasapriyan

**Affiliations:** CAS Key Laboratory of Nanosystem and Hierarchical Fabrication, CAS Center for Excellence in Nanoscience, National Center for Nanoscience and Technology Beijing 100190 China vsripriyan@nanoctr.cn vsripriyan@icloud.com; University of Chinese Academy of Sciences Beijing 100049 China

## Abstract

The material demand for metal organic framework (MOF) production necessitates advance in their expedient and scalable synthesis that can operate at room temperature which would be pivotal for chemical industry. Toward that end, M-Hoba, where M = divalent metal acetate and Hoba = 4,4′-oxybis(benzoic acid), can now be prepared in minutes *via* a controlled dissolution–crystallization route with divalent metal acetate as a precursor. MOFs prepared by this strategy were highly crystalline. This synthetic method was also applied successfully to the synthesis of a MOF-5 series (Cu) and Cu-BTC to highlight its generality. This method can move traditional MOF synthesis from the laboratory scale to the industrial scale.

## Abstract 2

The material demand for metal organic framework (MOF) production necessitates advances in their expedient and scalable synthesis at room temperature, which is pivotal for the chemical industry.

## Graphical abstract

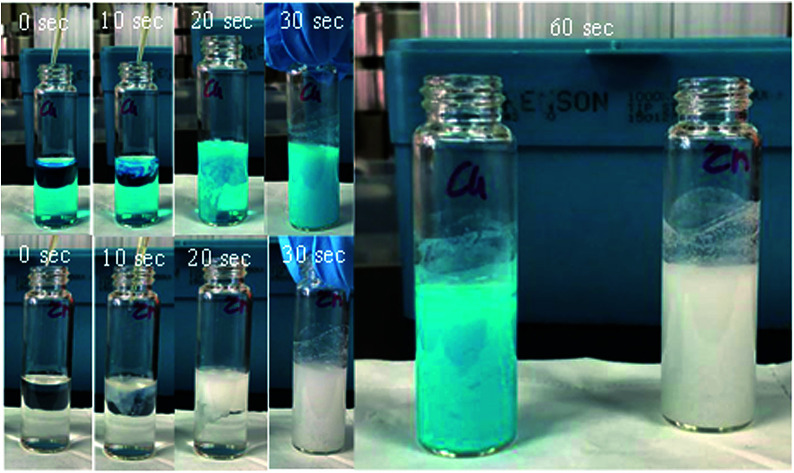

MOFs are porous materials synthesized from inorganic metal ions and organic ligands, with functional tunability, ultrahigh porosity and enormous internal large surface areas for active sites.^1–3^ Moreover, layered MOFs have highly accessible active sites on their surface, which could be ideal for catalytic applications.^4–9^ The industrial adoption of MOFs would be greatly improved through a scalable rapid synthesis method that can operate at room temperature, which would be pivotal for the chemical industry; it would require the careful selection of the metal precursor in terms of both the environmental impact and cost.^10,11^ MOFs are typically synthesized under harsh conditions, requiring high temperatures and pressures, which results in high production costs. The demands for metal–organic framework (MOF) materials for next-generation energy storage and conversion, in the fields of storage, purification, separation, and heterogeneous catalysis, necessitate advances in their expedient and scalable synthesis under ambient conditions and on short timescales.^12–17^ MOFs are generally synthesized *via* solvothermal or hydrothermal methods,^18^ which typically require high temperatures and pressures;^19^ however, until now, they have been some of the most widely used synthetic methods for preparing MOF materials from the milligram scale to a larger scale level. Recent reviews have considered scalable synthetic MOF production and commercialization efforts.^20^ Julien *et al.* highlighted the most important factors of concern during scalable and sustainable MOF synthesis.^21^

Miquel Gimeno-Fabra *et al.* demonstrated the first MOF synthesis production method using a countercurrent mixing reactor; in this they used preheated water at around 400 °C at the mixing point. The first material synthesized *via* this method was Cu-BTC, with reaction times of 3 to 12 h. This makes them of interest industrially, where they may engender novel approaches for energy applications and environmental remediation technologies.^22^ However, their instant mixing method requires very high temperatures and long timescales. Lorenzo Maserati *et al.* reported^23,24^ the rapid synthesis of an M-MOF-74 series from divalent metal oxides. This is the most rapid MOF synthesis.^25^ However, it's not applicable as a common method for the synthesis of other standard MOFs. In the laboratory, MOFs are synthesized on milligram scales, requiring high temperatures and multi-day reaction times with pressure vessels. Therefore, a scalable rapid synthesis method that operates at room temperature would be pivotal for the chemical industry to move from the milligram scale to a larger scale.^20,21,26–32^ Herein, we report MOF synthesis with divalent metal acetate as a precursor *via* a controlled dissolution–crystallization method in minutes. This doesn't require high temperatures, pressures, or long timescales. It's a simple and instant method for MOF synthesis, and also can be applied to particular standard MOFs.

In catalyst preparation where water can be utilized as a medium, the use of organic solvent is minimized at a ratio of 3 : 1 (water : DMF). To begin the accelerated pace metal acetate-to-MOF process (Fig. 1), the metal acetate was dissolved completely in water and filtered through a filter membrane (0.22 μm), followed by simply mixing in the organic linker (dissolved in DMF). This MA-to-MOF transformation was likewise observed *via* the naked eye, where the analogous liquid-to-solid transformation was observed over a time span of 0–60 s at room temperature (Fig. S1†). The SEM images and PXRD data in Fig. 2 and 3 show the transformations of the morphologies and crystallinities of the MOFs from 30 s to 1 h. The reaction was allowed to proceed until the liquid-to-solid transformation was complete (seconds to minutes, depending on the metal acetate). Fig. 4 and 5 show TEM images and elemental mapping, confirming the morphologies and uniform distributions of the metal nodes. Fourier transform infrared spectroscopy (FTIR) spectra of the MOFs (Fig. S5–S8†) show characteristic bands that are associated with organic ligands, water, DMF solvent, and copper coordination groups in the frameworks. The characteristic band at 1110 cm^−1^ shows the formation of C–O–Cu bonds. The band at 1666 cm^−1^ is assigned to the carbonyl group in DMF. Benzene ring bands are exhibited at 1578, 1501, 1399, 1156, and 1017 cm^−1^. The other bands are attributed to moisture or C–H bands in the fingerprint region. The characteristic XPS peaks of Cu 2p_3/2_ were observed at 934.8 eV, showing the existence of divalent Cu(ii) species in the MOFs. Interestingly, these reaction conditions were also applied successfully to the synthesis of standard MOFs, including the MOF-5 series (Cu) and Cu-BTC, (Fig. S3 and S4†) and further investigation regarding the scope of this method is currently under progress in the laboratory with structural modifications and other standard MOFs.

**Fig. 1:**
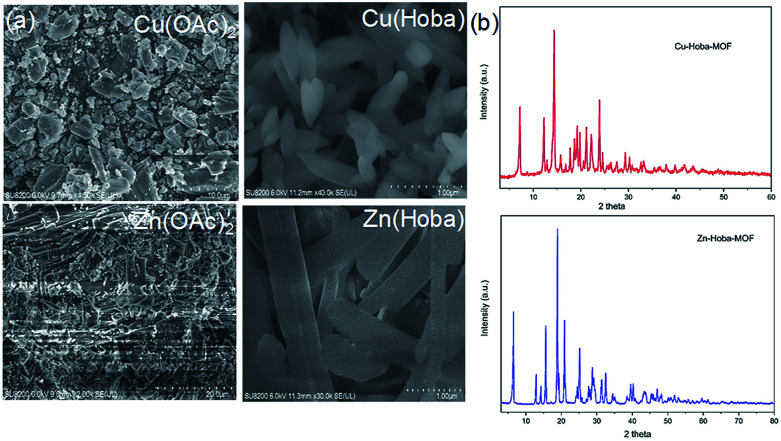
(a) SEM images of MA precursors (left column) and their respective reaction products with Hoba in water and DMF. (b) PXRD patterns of the products synthesized in one minute.

**Fig. 2:**
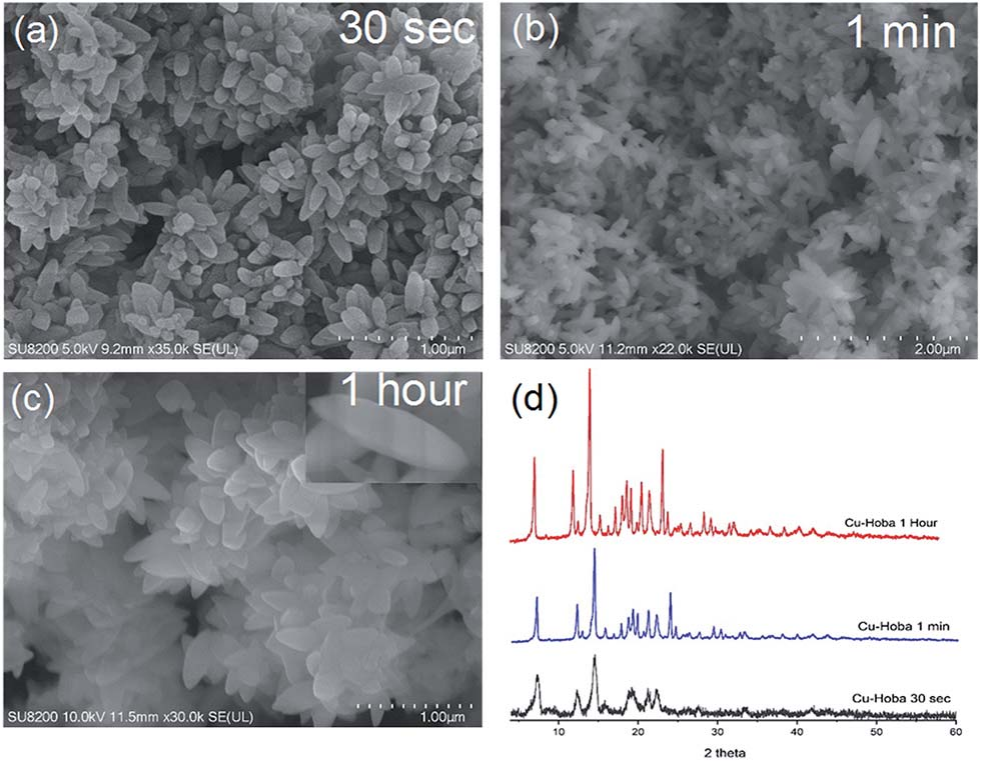
Scanning electron microscopy (SEM) images (a–c) of Cu-Hoba and (d) PXRD patterns showing the instant formation of Cu-Hoba MOF over timescales from 30 s to 1 h.

**Fig. 3:**
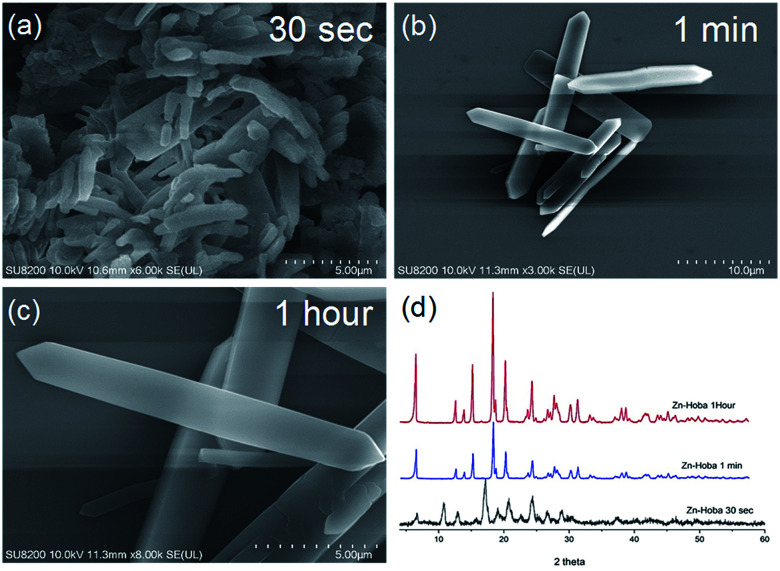
Scanning electron microscopy (SEM) images (a–c) of Zn-Hoba and (d) PXRD patterns showing the instant formation of Zn-Hoba MOF over timescales from 30 s to 1 h.

**Fig. 4:**
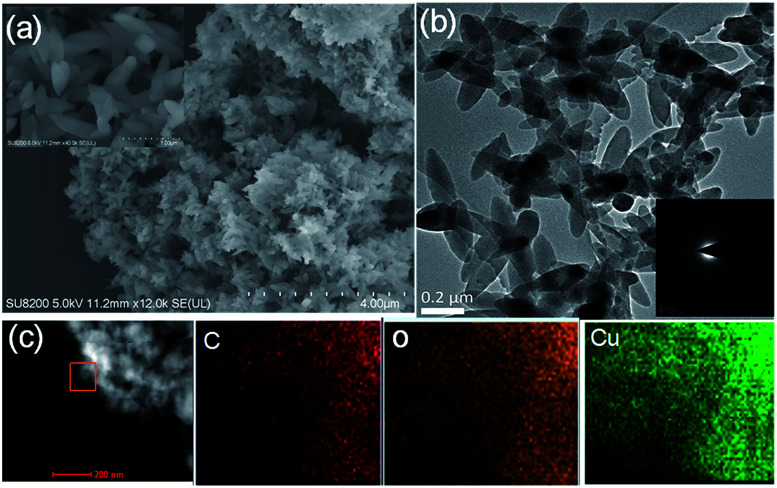
(a) Scanning electron microscopy (SEM) images of Cu-Hoba; inset: a larger scale image. (b) Transmission electron microscopy (TEM) images of Cu-Hoba; inset: the SAED (selected area electron diffraction) pattern. (c) A high-angle annular dark field scanning transmission electron microscopy (HAADF-STEM) image and the corresponding energy-dispersive X-ray spectroscopy (EDS) elemental mapping, after synthesis in 1 h.

**Fig. 5:**
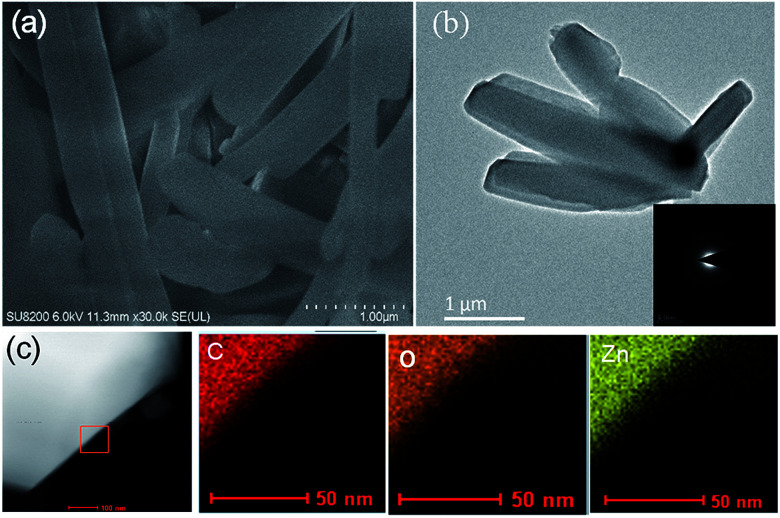
(a) A scanning electron microscopy (SEM) image of Zn-Hoba. (b) A transmission electron microscopy (TEM) image of Zn-Hoba; inset: the SAED (selected area electron diffraction) pattern. (c) A high-angle annular dark field scanning transmission electron microscopy (HAADF-STEM) image and the corresponding energy-dispersive X-ray spectroscopy (EDS) elemental mapping, after synthesis in 1 h.

The selective oxidation of alcohols to aldehydes plays a vital role in the chemical industry. In particular, benzaldehyde synthesis from benzyl alcohol attracts tremendous interest because it's a raw material for other organic compounds in industry, and it is also an intermediate in agrochemical and drug synthesis. Several studies have reported the catalytic oxidation of benzyl alcohol to benzaldehyde using different catalysts and oxidants. When commercial Cu(OAc)_2_ and CuCl_2_ were used as catalysts, negligible benzaldehyde was produced (Table 1). First, we screened the reaction to evaluate different parameters, like the oxidant, solvent, and catalyst. From the screening tests, we conclude that the selective oxidation of benzyl alcohol to benzaldehyde could occur with TBHP as an oxidant and DMF as a solvent, catalyzed by the Cu-Hoba MOF. The recyclability was checked over five consecutive cycles with no apparent loss of catalytic activity (Fig. S9 and 10†).

**Table 1:** BzOH oxidation with various catalysts under standard conditions^a^

No	Catalyst	Conversion
1	Cu acetate	2%
2	Cu chloride	4%
3	Cu-Hoba	60.2%

a Reaction conditions: benzyl alcohol: 0.5 ml; catalyst: 50 mg; DMF: 1 ml; oxidant: 2 ml TBHP; 30 °C; 18 h.

In conclusion, we expand our new method of MOF discovery into the field of catalysis. The demand for the expedient and scalable production of MOF materials is vital for next-generation technologies. We focus here on the basic chemistry that expedites MOF formation from a metal acetate precursor *via* a dissolution crystallization method. There is lots to learn from this new method, from the milligram to the molecular scale, and our understanding of this scalable minutes-long synthesis method is an important guidepost on the path to commercial MOF production. This synthesis can be done at room temperature, without any additional pressure, in a few minutes. Therefore, we hope that this MOF production method can replace commercial hydrothermal or solvothermal methods in industrial plants. Also, the synthesis and catalysis are done at room temperature. Therefore, this method can replace the noble metal catalysts used for alcohol oxidation reactions in industrial plants.

## Conflicts of interest

There are no conflicts to declare.

## Supplementary Material

NA-001-C9NA00350A-s001

NA-001-C9NA00350A-s002
