# Supplementary material for: Minute/instant-MOFs: versatile, high quality, ultrafast, scalable production at room temperature
Source: Nanoscale Adv. 2019 Aug 2;1(9):3379–82. doi: 10.1039/c9na00350a (PMC9417639; doi:10.1039/c9na00350a)
Supplement: NA-001-C9NA00350A-s001 [file NA-001-C9NA00350A-s001.pdf]

Electronic Supplementary Material (ESI) for Nanoscale Advances.
This journal is © The Royal Society of Chemistry 2019

Part S1. Chemicals and Reagents

Copper (II) acetate monohydrate, Zinc acetate-dihydrate, by Aladdin. 1,3,5-benzenetricarboxylic acid (H_3BTC, 95wt%), and BDC was purchased from Sigma-Aldrich (China). 4,4′-Oxybis (benzoic acid) (98 %) by Aladdin. The deionized water used in our experiments was obtained from the Milli-Q System. Other analytical grade solvents including DMF, Ethanol, Methanol were supplied by Beijing Chemical Reagent Company (China). All the chemicals were used without further purification.

Part S2. General Characterization

Scanning electron microscopy (SEM) measurement was performed on a Hitachi SU8200 scanning electron microscope at 6.0 kV. Transmission electron microscopy (TEM) and high-resolution TEM (HRTEM) imaging and High angle annular dark field scanning transmission electron microscopy (HAADF-STEM) imaging was carried out using Tecnai G_2 F20 S-TWIN at 200 kV. Powder X-ray diffraction (XRD) patterns were recorded on D/MAX-TTRIII (CBO) with Cu Kα radiation (λ = 1.542 Å) operating at 40 kV. X-ray photoelectron spectroscopy (XPS) spectra were performed by an ESCALAB 20 Xi XPS system, where the analysis chamber was 1.5×10^{-9} mbar and the size of X-ray spot was 500 μm. The chemical structure of different MOFs was investigated by FT-IR spectroscopy (Nicolet Nexus 670, USA).

General synthesis method

Metal acetate (0.5 mmol) were dissolved in distilled water and H_2oba (4,4′-Oxybis (benzoic acid) (1 mmol) were dissolved in DMF, Then the DMF solution was mixed by rapid solvent mixing method to water solution to get instant MOFs as shown in the Fig 1. Then centrifuged and washed with DMF and MeOH, dried at room temperature. Cu acetate 100 mg dissolved in 3 ml of water and 258 mg of (4,4′-Oxybis (benzoic acid) dissolved in 1 ml of DMF. Zn acetate 87.6 mg dissolved in 3 ml of water and 258mg of (4,4′-Oxybis (benzoic acid) dissolved in 1 ml of DMF.

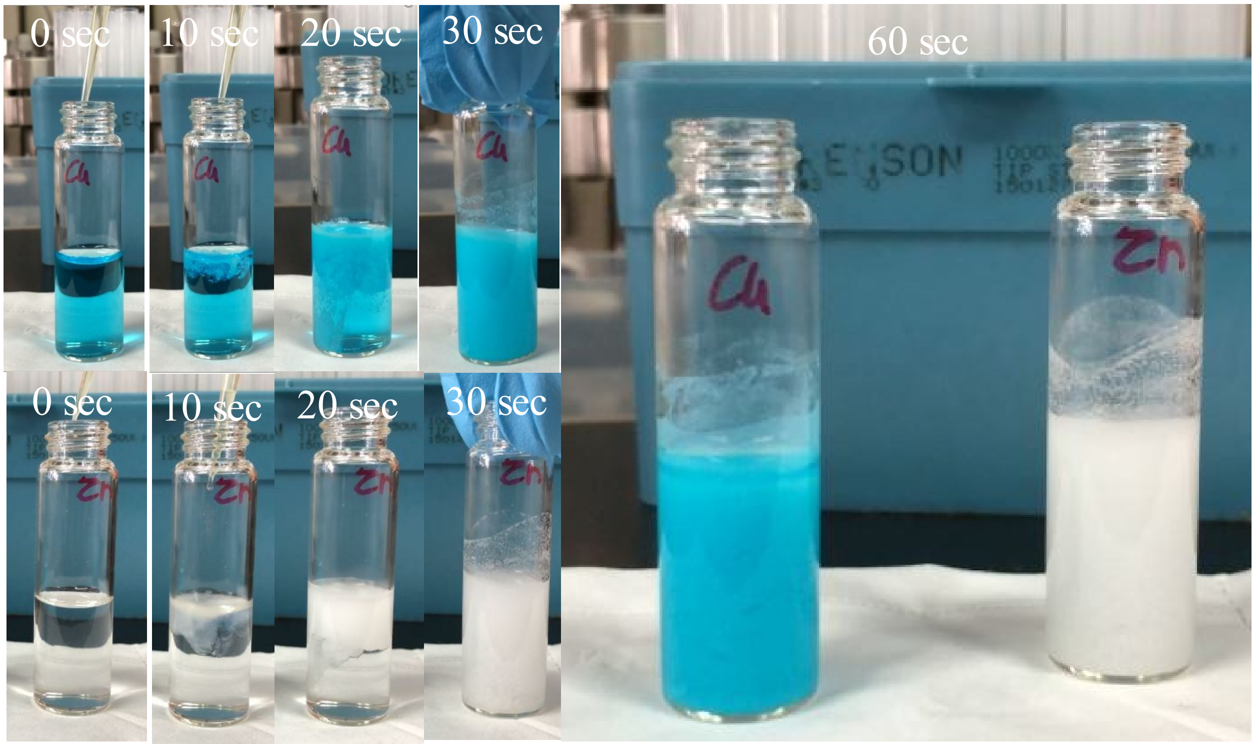

Fig S1 Minute-MOF Concept and Realization shows the formation of instant M (Cu or Zn) - Hoba MOF from 0 sec to 60 seconds.

Gram scale synthesis method

Cu acetate 1g dissolved in 30ml of water and 2.58g of (4,4′-Oxybis (benzoic acid) dissolved in 10 ml of DMF. Zn acetate 876 mg dissolved in 30 ml of water and 2.58g of (4,4′-Oxybis (benzoic acid) dissolved in 10 ml of DMF.

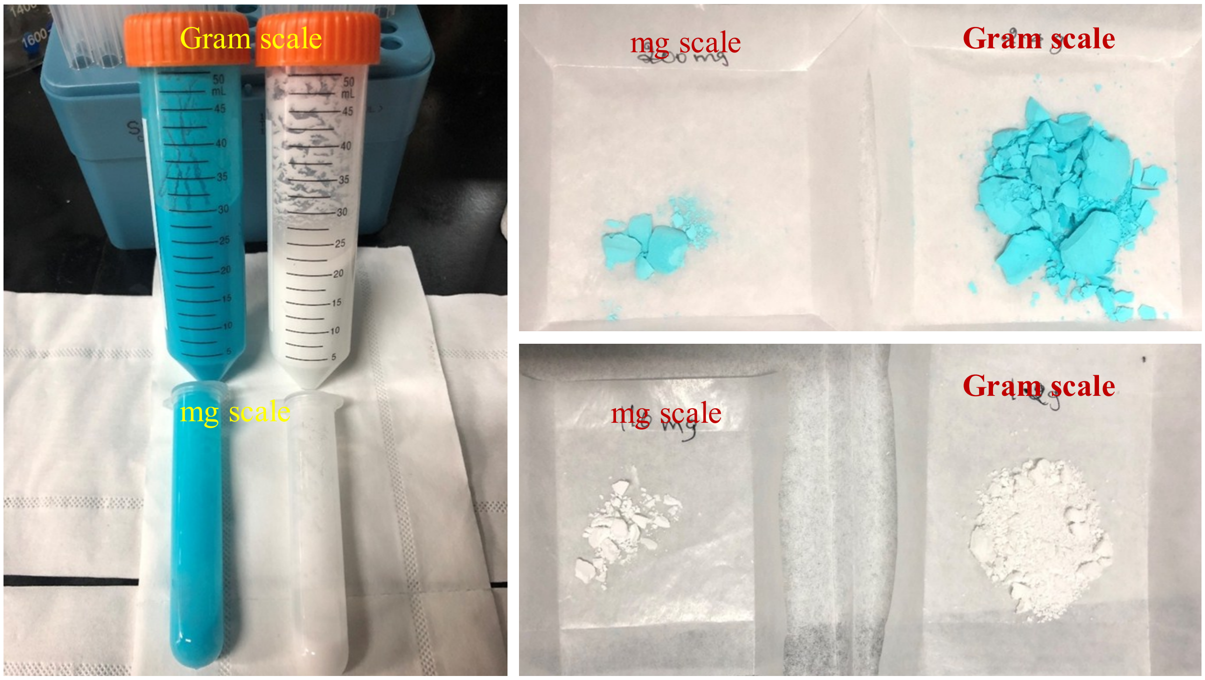

Fig. S2 **S**calable synthesis of instant M (Cu or Zn) -Hoba MOF from milligram to gram scale in a minute to 1h.

MOF-5 (Cu)

1. Cu acetate 100 mg dissolved in 3 ml of water and 166 mg of Benzene-1,4 dicarboxylic acid dissolved in 1 ml of DMF.

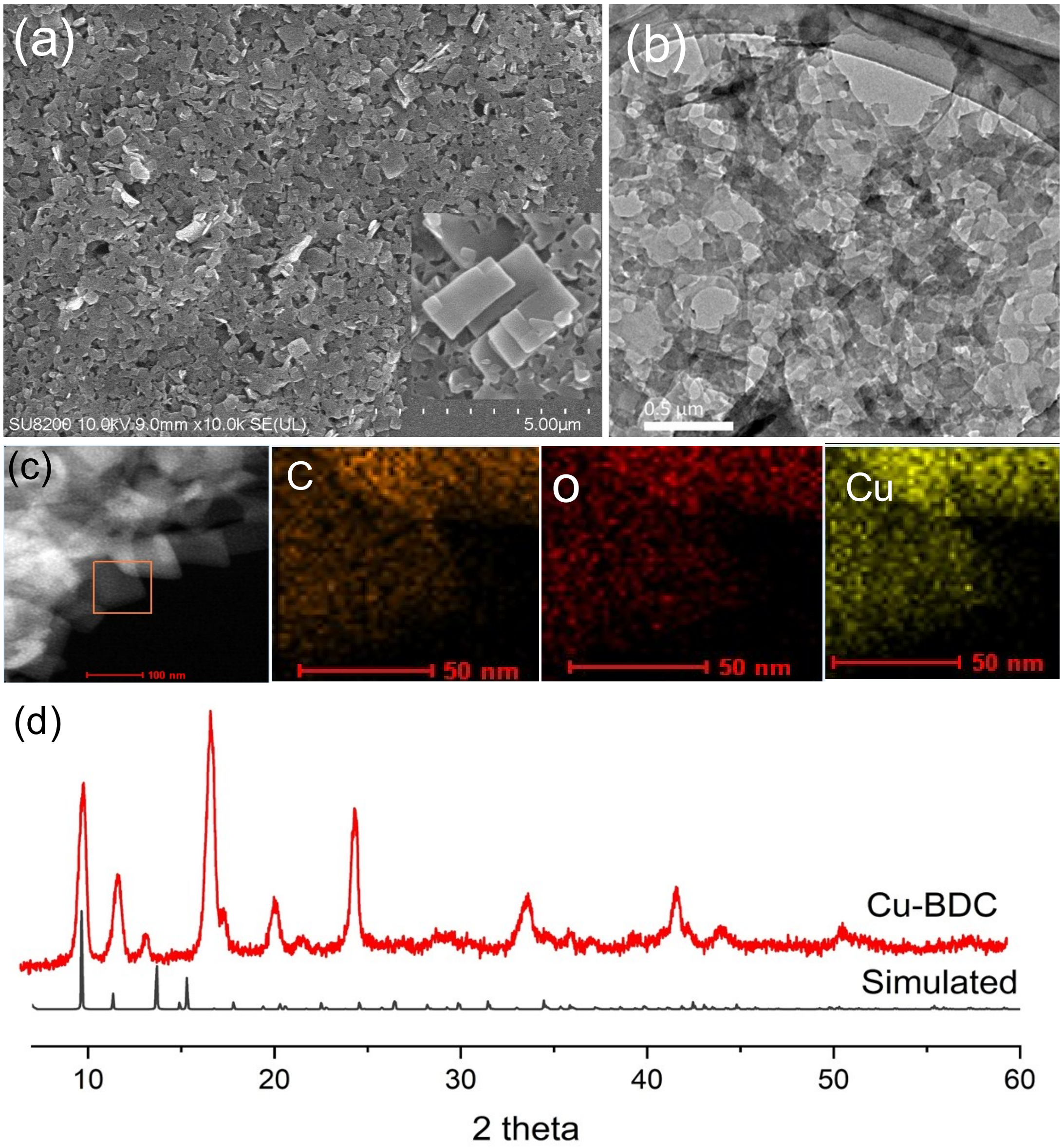

Fig. S3 (a) SEM Large-Scale image of Cu-BDC, insert magnified SEM images (b) TEM image and (c) High-angle annular dark field scanning transmission electron microscopy (HAADF-STEM) image and corresponding energy-dispersive X-ray spectroscopy (EDS) elemental mapping. (d) PXRD of synthesized CuBDC.

Cu-BTC

Cu acetate 100 mg dissolved in 3 ml of water and 210 mg of 1,3,5-tricarboxylic acid dissolved in 1 ml of DMF.

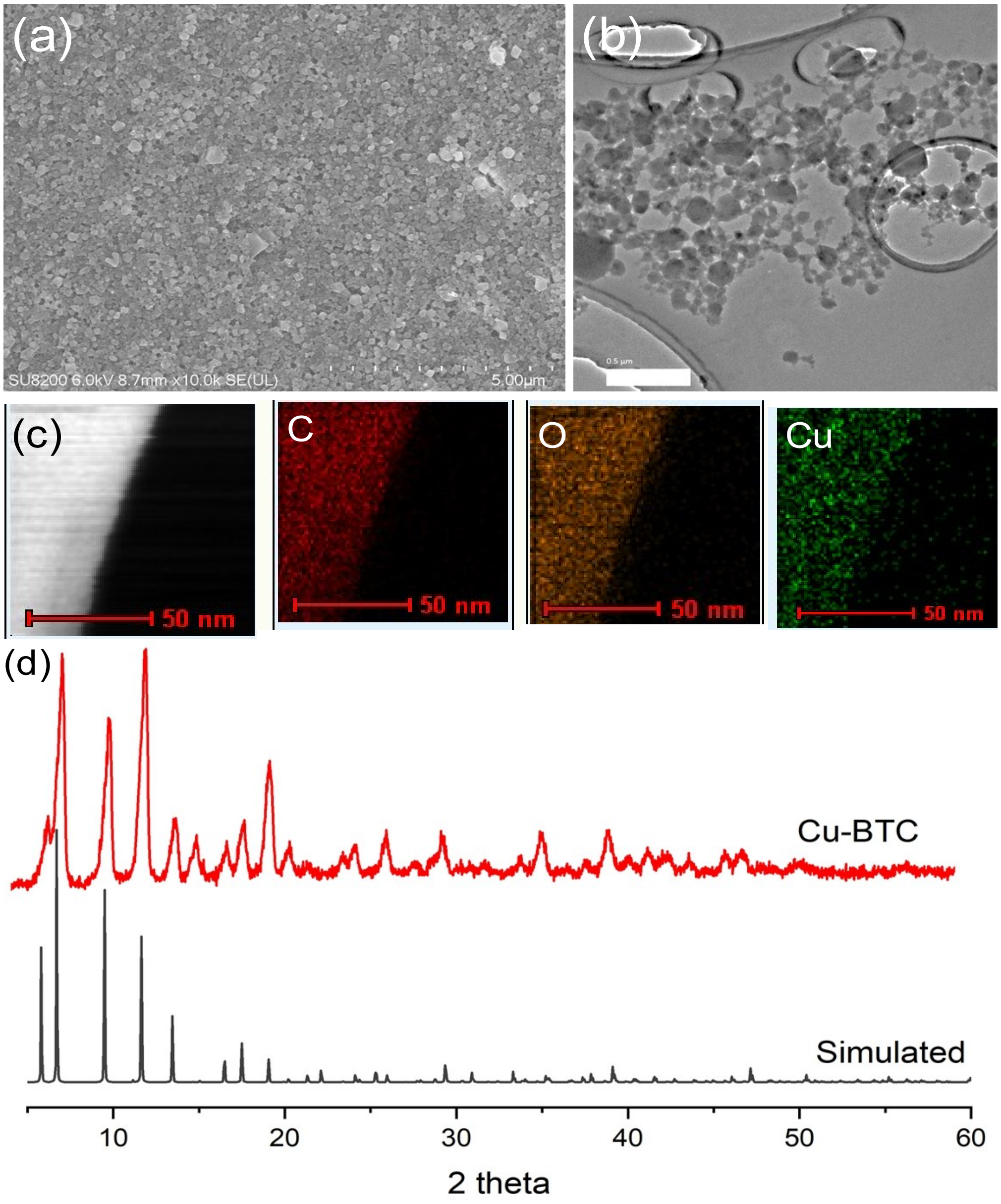

Fig. S4 (a) SEM Large-Scale image of Cu-BTC (b) TEM image and (c) High-angle annular dark field scanning transmission electron microscopy (HAADF-STEM) image and corresponding

energy-dispersive X-ray spectroscopy (EDS) elemental mapping. (d) PXRD of syntheised CuBTC.

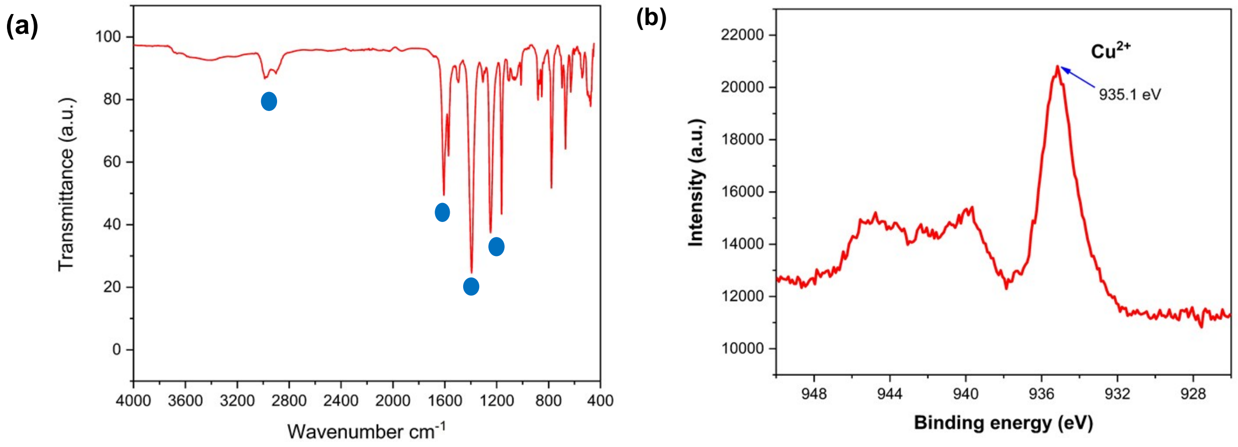

Fig. S5 Characterizations of Cu-Hoba MOF (a) FTIR spectrum, in which the symbols represent the specific organic groups discussed in the main text. (b) XPS spectrum of Cu $2p_{3/2}$ in Cu-Hoba.

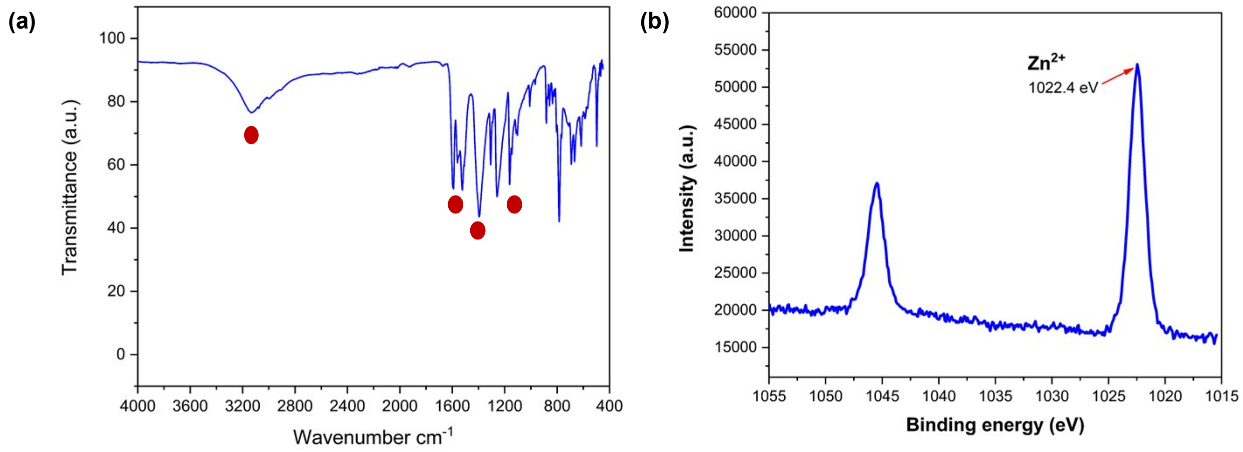

Fig. S6 Characterizations of Zn-Hoba MOF (a) FTIR spectrum, in which the symbols represent the specific organic groups discussed in the main text. (b) XPS spectrum of Zn $2p_{3/2}$ in Zn-Hoba.

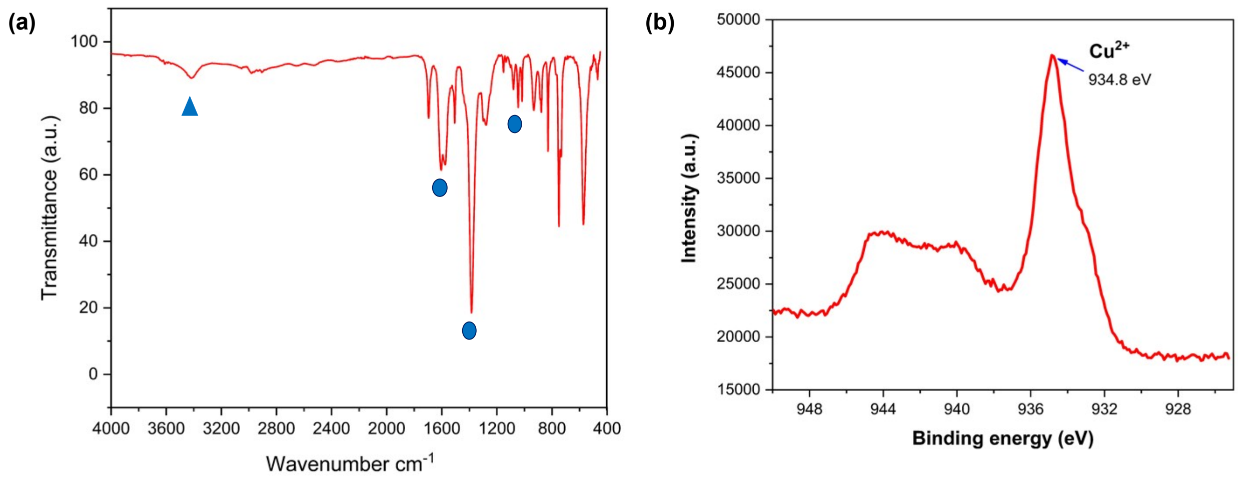

Fig. S7 Characterizations of Cu-BDC MOF (a) FTIR spectrum, in which the symbols represent the specific organic groups discussed in the main text. (b) XPS spectrum of Cu $2p_{3/2}$ in Cu-BDC.

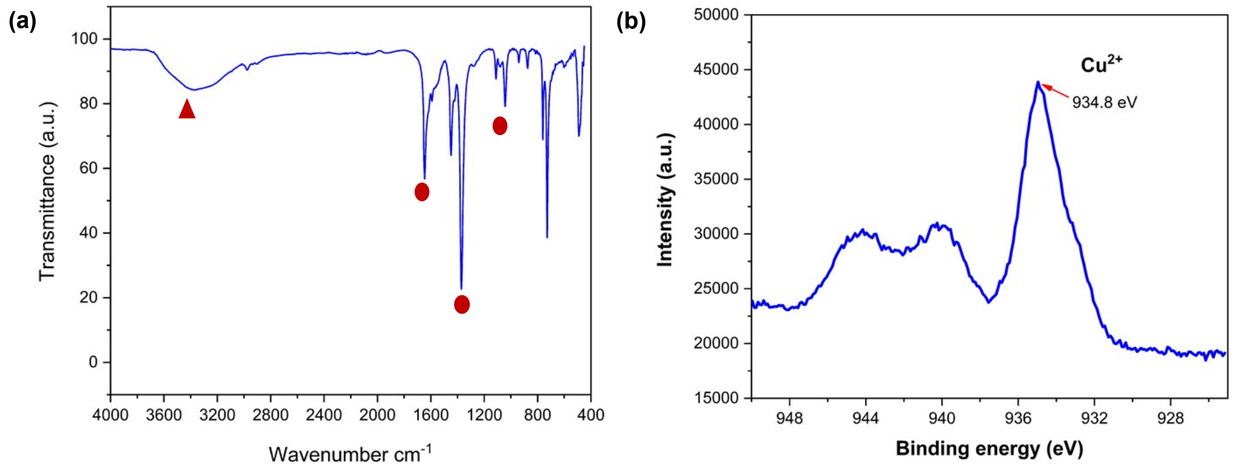

Fig. S8 Characterizations of Cu-BTC (a) FTIR spectrum, in which the symbols represent the specific organic groups discussed in the main text. (b) XPS spectrum of Cu $2p_{3/2}$ in Cu-BTC.

BzOH oxidation with various oxidants under standards conditions.

No	Oxidant	conversion
1	Air	1% ≤
2	H_2O_2	6.20 %
3	TBHP	8 %

Reaction condition: Benzyl alcohol (1mL), catalyst: 20mg, solvent: 1ml DMF, oxidant at 30 ℃, 3h.

BzOH oxidation with various solvents under standards conditions.

No	Solvent	conversion
1	Neat	1% ≤
2	water	6.6 %
3	Ethanol	6 %
4	DMF	18 %

Reaction condition: Benzyl alcohol (1mL), catalyst: 20mg, oxidant: 1ml TBHP, at 30 ℃, 3h.

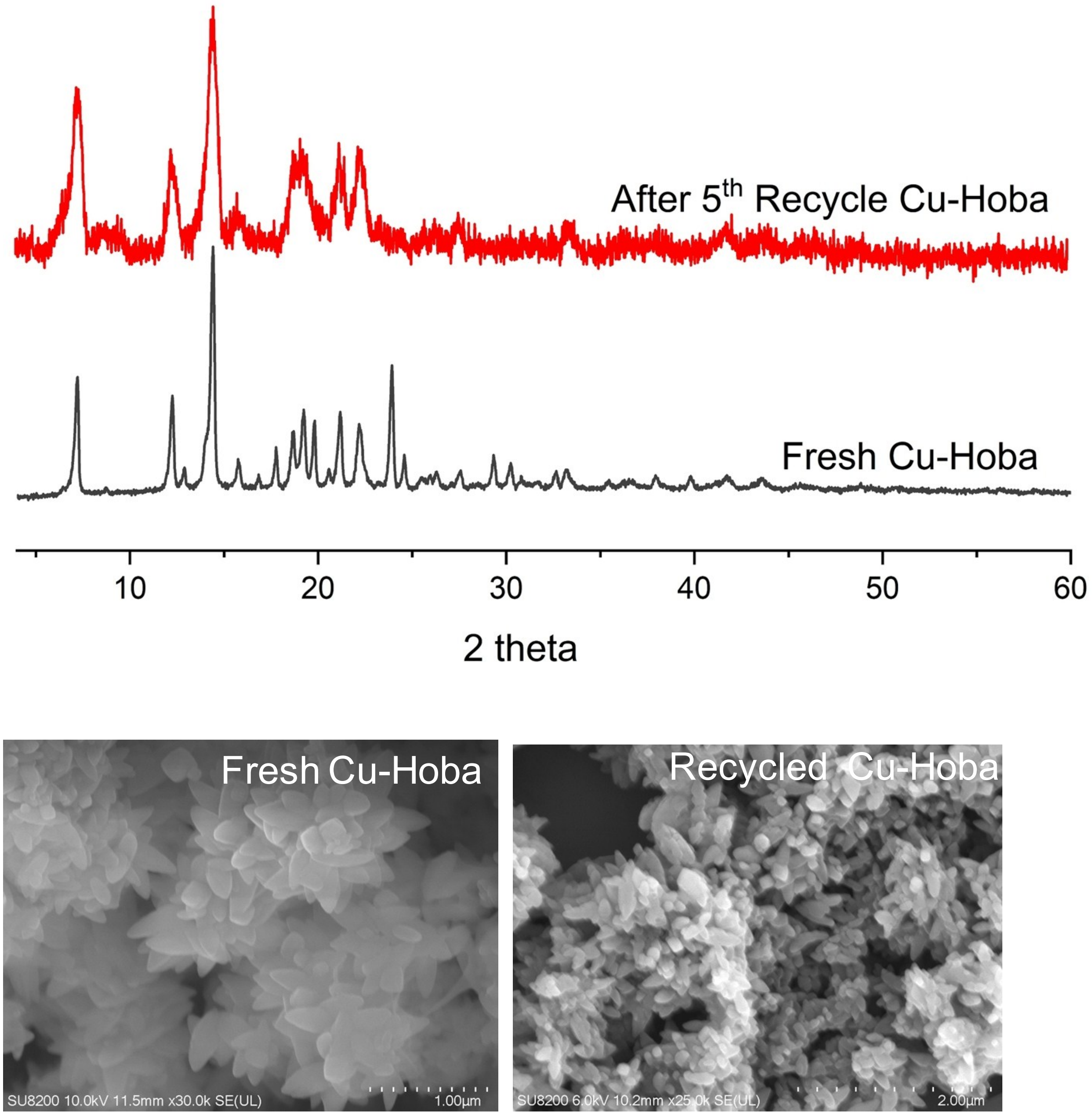

Fig. S9 (a) recovered catalyst PXRD (b) SEM image of Fresh and recovered Cu-Hoba.

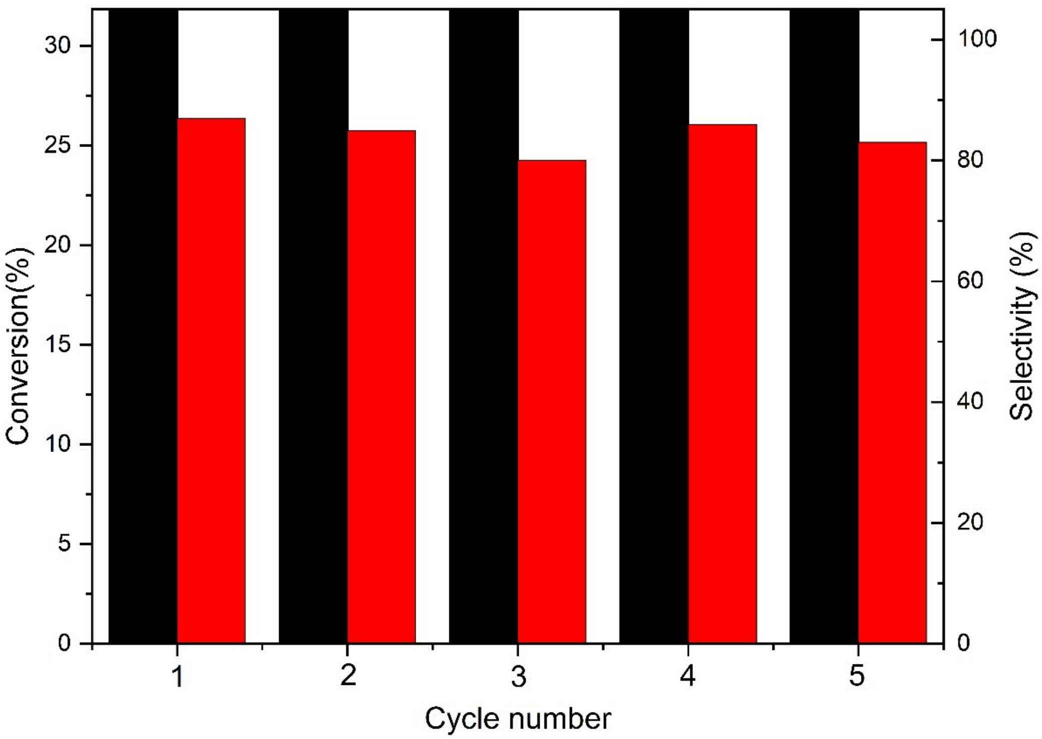

Fig. S10 Stability test of Cu-Hoba
